# Socioeconomic disparities and inequality of mass sports participation: Analysis from Chinese General Social Survey 2010–2018

**DOI:** 10.3389/fpubh.2023.1072944

**Published:** 2023-02-09

**Authors:** Haoyu Dong, Ying Wang, Wen Li, Jennifer Dindin

**Affiliations:** ^1^College of Teacher Education, University of Cordilleras, Baguio, Philippines; ^2^School of Public Policy and Administration, Chongqing University, Chongqing, China

**Keywords:** mass sports participation, inequality, CGSS, China mass sports, socioeconomic disparities

## Abstract

**Background:**

With industrialization, urbanization, and modernization, mass sports have entered people's daily lives to maintain their health status. However, less attention has been paid to the heterogeneity and inequality of access to mass sports, especially in developing countries. This study aims to analyze the factors that affect mass sports participation in developing countries represented by China, and explain the changing trends and inequality in the class differentiation and mobility of public sports participation.

**Methods:**

The study selected the Chinese General Social Survey (CGSS) data in 2010 and 2018 as the research samples, and used an ordered Probit model and sub-sample regression to analyze the factors and trends of Chinese residents' mass sports participation and the influencing factors. By stratified three-stage probability sampling, the study obtained 4,940 valid responses, including 1,014 in CGSS 2010 and 3926 in CGSS 2018.

**Results:**

First, in terms of social factors, urban residents have a higher frequency of sports participation than rural residents. Second, regarding family factors, residents with higher social classes are more likely to participate in sports than those with lower social classes. Third, in terms of self-induced factors, the elderly are more motivated to exercise than the young. Residents with public-sector jobs, high incomes, and higher education levels are keener to participate in sports. Fourth, residents' mass sports participation rate has generally shown an upward trend over time. Fifth, with time changes, the sports participation rate varies between urban and rural areas, between ethnic minorities and Han ethnic, between old and young age groups, and between higher and lower education levels will continue to shrink, but differences between social classes will further increase over time.

**Conclusions:**

Our analysis demonstrated that hidden inequality existed in accessing mass sports participation in developing countries, and the self-induced characteristics were significantly correlated with the quality of sports participation. Future public sports policies should address the inequity to ensure equal access to affordable qualified personal mass sports.

## 1. Introduction

With the improvement of social and economic levels, human values gradually surpass the pursuit of a single economic dimension, and pay more attention to multi-dimensional experiences such as social, psychological and physical health (1). When health sociologists observe changes in perceptions, they find that socioeconomic factors such as social status, education and income level of individuals are closely related to the attainment of physical health (2–8). The inequality of sports participation caused by social class distinction has attracted scholars' attention.

From the perspective of sports sociology, sports access or mass sports participation can be regarded as one of the most common social phenomena in modern society and one of the crucial ways for ordinary members of society to achieve socialization (9). Earlier, Kenyon (9) and other scholars refined the meaning of sports participation. He believed that sports participation covered the level of sports cognition, emotional tendency, direct participation, and indirect participation related to social class characteristics (10). Therefore, under this background, some scholars believe that the inequality of mass sports participation is due to the existence of class divisions. The way of sports participation, as a means of class division, is not only reflected in the choice of sports events, but also limited sports participation (10–13). With the development of democracy, sports participation is no longer restricted by class divisions, but by sports itself, which makes the inequality of sports participation appear in another form (14, 15). For example, the upper class or more affluent group excludes others through expensive consumption or strict membership in golf and rowing clubs, while the working class is attracted by exciting sports such as boxing, wrestling, and dog running (16). At the same time, a strict distinction has been made between professional and amateur athletes, which does not release the inequality of participation (14, 17).

Up to now, sports participation has been gradually decoupled from the political class. Economic or willingness analyzes of new technologies are beginning to be applied to sports participation (18, 19). The development of blockchain and other technologies has effectively protected personal sports and health data (20, 21). Sports participation bears more weight on individual development and family health living. Under this trend, sports participation is divided more finely. On a global scale, scholars have divided sports participation into mass and elite/professional sports participation (22). More specifically, three forms of sports participation have been formed: competitive sports participation, school sports participation, and mass sports participation (23–25). Among them, competitive sports focus on stimulating human beings. To maximize the potential of physical fitness and psychological endurance, it emphasizes starting from all human beings, regardless of ethnicity and race, so when we discuss participation in competitive sports, we emphasize individual-level talent. Schools' physical education is a form of physical education related to school sports participation (23, 24, 26). It pays more attention to the methods of physical education, the position and development prospects of physical education in quality education, and the discussion of school sports participation pays more attention to the influence of the social level. Mass sports participation is the generalization of residents' daily exercise habits and amateur sports acquisition, which is usually closely related to lifestyle, healthy exercise, etc., so the discussion on mass sports participation needs to consider both the individual level and the social level (27).

Based on the above classification, this article chooses to focus on sports participation in mass sports. Compared with competitive and school sports, mass sports are more closely related to each of us. Meanwhile, due to economic improvement, residents are paying more and more attention to healthy lifestyles, and mass sports can include as many samples as possible. Research on mass sports participation can better reflect the changes in mass exercise methods and the development of health policies.

Hence, the research will start with the specific factors that affect mass sports participation and answer two progressive questions:

(1) Does the inequality of mass sports participation in developing countries (China) exist? If it exists, what are the specific influencing factors; if it does not, clarifies the presentation form of mass sports participation.
(2) With the changes of the times, what kind of changing trend does mass sports participation in developing countries show? Has inequality improved amid this trend?

By sorting out residents' sports participation tendencies, we find that social structures, such as class, status, prestige, power, etc., affect the socialization of sports participation to a certain extent (28–31). In fact, there have been discussions of sports participation from the perspective of social stratification theory. In the late twentieth century, scholars noticed that sports participation as a means of social stereotyping was widely used throughout society and played a role as a maker of social inequality in social stratification (28, 32). Giddens (33) once stated that the liberation of the individual is the freedom obtained by breaking free from the constraints of inequality. Inequality theory has become a direction that public sociologists and scholars engaged in a social policy called on government authorities to promote public participation in sports (17, 34–36). Sports have become a powerful weapon for reform in project promotion and sports facility construction.

In sociology, scholars have also linked public sports participation with social structure and individual agency along the perspective of social stratification theory (14, 32, 37–39). Social structure, that is, we usually understand various external environmental factors that are independent of the individual but can restrict the individual, while individual agency emphasizes the individual's ability to independently choose and carry out actions (40). It is undeniable that the social structure is independent of the individual and is indeed a factor that cannot be ignored in the changing times. The individual initiative will also check, balance, and influence the social structure. However, this distinction divides the individual and society. In fact, the distinction between the micro-level and the macro-level does not have such a large span. Similarly, the social structure at the macro level does not always directly act on individuals through external factors, but often looks for some intermediaries, such as government organizations (governments) and some public profit organizations (hospitals) that we cannot ignore.

Scholars' research in recent years has also found that the family has a non-negligible influence on the behavior and habits of adults, and the way family upbringing will affect the individual's lifestyle in adulthood (2, 39, 41, 42). Therefore, in order to ensure the scientific nature of the research, this study believes that discussions such as family annual income and family social class also need to be included in the frame design.

Specifically, in this study, the author attempts to classify the factors that affect mass sports participation into social factors, family factors and self-induced factors, and accordingly put forward the following hypotheses:

**H1: Inequality in mass sports participation exists in developing countries (China)**.

*H1a: Social factors have a significant impact on mass sports participation*.

*H1b: Family factors have a significant impact on mass sports participation*.

*H1c: Self-induced factors have a significant impact on mass sports participation*.

**H2: As times change, inequality in mass sports participation in developing countries will improve**.

## 2. Methods

### 2.1. Setting and data resources

The data in this paper comes from the Chinese General Social Survey (CGSS). CGSS is the earliest national, comprehensive and continuous academic survey project in China, implemented by the China Survey and Data Center of Renmin University of China. Since 2003, the project has basically guaranteed to conduct continuous cross-sectional surveys on more than 10,000 households in various provincial units in mainland China once a year. 2003–2008 is the first phase of the CGSS project. A total of 5 annual surveys were completed (exclude 2007), and 5 sets of high-quality annual data were produced. 2010-2019 is the second phase of the CGSS project. As of the article writing, 7 annual survey data (2010, 2011, 2012, 2013, 2015, 2017, 2018) have been released, the latest year of data available is 2018.

This article explores Chinese residents' sports participation and its influencing factors. Considering that with the evolution of the times, Chinese citizens' attitudes toward physical participation have changed, this article will also attempt to deeply explore the changing trends of the effects of various influencing factors. The authors selected the CGSS data in 2010 and 2018 as the research samples. CGSS 2010 and 2018 contain several parts, including core modules (basic information), class consciousness, social stratification, income and consumption, religion, environment, and health. After removing missing values and outliers, the study obtained 4940 valid observation samples, including 1014 in CGSS 2010 and 3926 in CGSS 2018.

### 2.2. Sampling method

#### 2.2.1. Stratified three-stage probability sampling

The CGSS is a national large-scale survey project which targets all urban and rural households in 31 provinces, autonomous regions, and municipalities directly under the Central Government (excluding Hong Kong, Macao, and Taiwan). CGSS 2010 and 2018 adopt stratified three-stage probability sampling. As shown in Table 1, the sampling units of each stage are slightly different.

**Table 1:** Stratified three-stage probability sampling.

	**Primary sampling unit (PSU)**	**Secondary sampling unit (SSU)**	**Third stage sampling unit**
Mandatory layer	Street	Neighborhood committee	Households
Lottery layer	District, county-level city, county	Neighborhood committee, village committee	Households

For the mandatory layer, choosing streets as the primary sampling unit can refine the sampling frame and make the sample points relatively scattered, which is conducive to collecting general information and avoids sample bias due to too thick a sampling frame. For the selection layer, considering that there are many districts, county-level cities, and counties in the whole country, it is more appropriate to use them as the primary sampling unit.

Based on past survey experience, the target sample size for the survey is set at 12,000 households, of which 2,000 are compulsory and 10,000 are selected. The sample size allocations covered in the subsequent sections are all based on the target sample size.

#### 2.2.2. Sample size

For the mandatory layer, the total sample size is 2,000 households. Specifically, 40 primary sampling units (streets) are selected, and two secondary sampling units (neighborhood committees) are chosen from each primary sampling unit (PSU). The third stage of sampling contains 25 households, which were selected from SSU.

For the selection layer, the total sample size is 10,000 households. Specifically, 100 PSUs (districts, county-level cities, counties) are selected, and 4 SSUs (neighborhood committees, village committees) are chosen from each PSU. Each neighborhood committee (village committee) selects 25 households.

Hence, a total number of 140 PSUs and 480 SSUs were drawn in this survey.

Considering that the answer rate in the survey is difficult to reach 100%, this plan adopts the method of expanding the sample size by using the expansion coefficient to enlarge the sample size of the third stage. According to the survey experience in previous years, due to various reasons, the answer rate of residents in the municipal districts of developed cities is 50%. That is, the expansion coefficient is around 2, so 50 households are selected from each second-level unit in the mandatory layer, and the contact sample size of this layer is expanded to 4,000.

The response rate of the residents in the selected layer is higher than that of the mandatory layer. In general, based on the experience in the previous year, the response rate of urban residents is around 65%, and the response rate of rural residents is higher than that of urban residents, roughly about 85%. Therefore, for the selection layer, 38 households were selected for each neighborhood committee, and 30 households were selected for each village committee.

This survey's final contact sample size is 17,664, of which 4,000 are mandatory, and 13,664 are selection. In the mandatory layer, when the number of primary and secondary units remains unchanged, the number of contact samples in each secondary unit increases to 50 households. In the selection layer, the number of primary units is 100, and the number of secondary units in each primary unit is 4. The contact sample size in each sampling neighborhood committee was expanded to 38 households, while in each sampling village committee was expanded to 30 households. Therefore, the contact sample size of the selection layer was 13,664, of which 7,904 were urban residents and 5,760 were rural residents.

### 2.3. Variable selection

#### 2.3.1. Dependent variable: Sports participation

The dependent variable in this paper is residents' sports participation. Unlike the previous studies, which defined sports participation as a dummy variable—whether or not to participate in physical exercise; this study believes that the previous method limits the study to qualitative analysis. In fact, there are also significant differences in the frequency of participation among groups that participate in physical activity. Therefore, this article will focus on the number of participants. For example, taking the 2010 questionnaire as an example, this research selects the following questions:

“*B4: How many times a week do you do physical exercise for at least 20 minutes or more? Here refer to those exercises that make sweat or breathe faster.”*

According to the answer, define the dependent variable sports participation and set the five frequencies of “I do not exercise”, “several times a year or even less”, “several times a month”, “several times a week” and “exercise every day” as 1, 2, 3, 4, 5. Similarly, in the 2018 questionnaire, the number of specific exercises was classified as the above five frequencies for assignment.

#### 2.3.2. Independent variable

Considering the diversification of factors influencing sports participation, whether or not to participate in physical exercise and the frequency of participation are often the result of the combined action of multiple factors. The past literature introduces a series of factors that may affect the dependent variable as independent variables from three aspects: social factors, family factors, and self-induced factors. (1) Social factors include household registration, gender, and ethnicity; (2) Family factors include family income and social class of the households at the age of 14; (3) Self-induced factors include age, occupation, individual income, education level, marital status, and religious beliefs. In addition, considering that the changes in the times may impact on sports participation, the study also introduces time virtual variables; the specific variable selection and assignment are shown in Tables 2–4.

**Table 2:** The measurement of the independent variable—social factors (CGSS 2018).

**Social factors**	**Question and assign**	**Frequency**	**Percentage**
Gender	**A2. What is your gender?**		
	female = 0	6,441	53.01
	male = 1	5,708	46.99
Household registration	**A18. Your current household registration status is**		
	Rural household registratio*n =* 1	6,994	54.70
	Urban household registratio*n =* 0	3,123	24.42
	Resident household (formerly rural household registration) = 0	1,010	7.90
	Resident household (formerly urban household registration) = 0	1,628	12.73
	Military status = None	5	0.04
	No account = None	5	0.04
	Other = None	22	0.17
Ethnicity	**A4.Your ethnicity is**		
	Ha*n =* 0	11,829	92.74
	Montgomery = 1	36	0.28
	Ma*n =* 1	97	0.76
	Hui = 1	235	1.84
	Zang = 1	7	0.05
	Zhuang = 1	145	1.14
	Wei = 1	3	0.02
	Other = 1	403	3.16

CGSS2010 and CGSS2018 have the same variable measurement methods in the related questions, the data of CGSS2010 will not be listed in detail.

**Table 3:** The measurement of the independent variable—family factors (CGSS 2018).

**Family factors**	**Question and assign**	**Frequency**	**Percentage**
A Family income	***A62.What was your family's total household income in 2017?*** 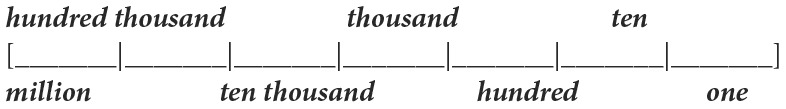		
	Family income = ln(a62 + 1)
Social class	**A4.When you were 14, what social class did you think your family was in?**		
	One point = 1	2,123	17.17
	Two points = 2	2,407	19.46
	Three points = 3	2,499	20.21
	Four points = 4	1,731	14.00
	Five points = 5	2,177	17.60
	Six points = 6	636	5.14
	Seven points = 7	301	2.43
	Eight points = 8	172	1.39
	Nine points = 9	39	0.32
	Ten points = 10	64	0.52
	Don't know = None	196	1.58
	Refuse to answer = None	21	0.17

CGSS2010 and CGSS2018 have the same variable measurement methods in the related questions, the data of CGSS2010 will not be listed in detail.

**Table 4:** The measurement of the independent variable- self-induced factors (CGSS 2018).

**Self-induced factors**	**Question and assign**		**Frequency**	**Percentage**
Individual income	***A8a.What was your total individual income for last year (2017)?*** 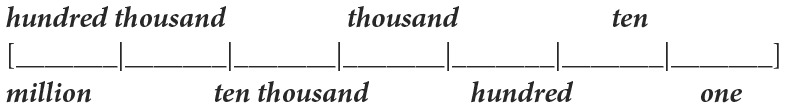			
		Income = ln(a8a+1)		
Age	**A3a. What is your date of birth? 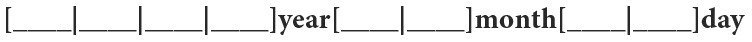 **		
		Age = 2018-a3a		
Marital status	**A69.What is your current marital status?**		
		Single = 0	1,204	9.91
		Cohabitatio*n =* 0	254	2.09
		First marriage with spouse = 1	8,882	73.11
		Remarried with spouse = 0	200	1.65
		Separated but not divorced = 0	68	0.56
		Divorce = 0	301	2.48
		Widowed = 0	1240	10.21
Education level	**A7a.Your current highest education level is**		
		Without any educatio*n =* 0	1,758	14.50
		Private schools = 6	77	0.64
		Primary schools = 6	2,612	21.54
		Junior high school = 9	3,260	26.89
		Vocational high school = 12	167	1.38
		General high school = 12	1,444	11.91
		Technical secondary school = 12	556	4.59
		Technical school = 12	51	0.42
		University Diploma (adult higher education) = 15	369	3.04
		University Associates (formal higher education) = 15	570	4.70
		Undergraduate (adult higher education) = 16	269	2.22
		Undergraduate (formal higher education) = 16	850	7.01
		Postgraduate and above = 19	142	1.17
Occupation	**A60j.The type of affiliation or company in which your most recent off-farm job was**		
		Party and government organs = 1	170	4.31
		Enterprise = 0	2,012	50.96
		Public institutions = 1	625	15.83
		Social groups, village/neighborhood committees = 0	132	3.34
		Unemployed/self-employed = 0	799	20.24
		Army = 0	34	0.86
		Other = None	95	2.41
		Don't know = None	70	1.77
		Refused to answer = None	11	0.28
Religious	**A5.What is your religion?**		
		No religio*n =* 0	10,852	89.32
		Buddhism = 1	533	4.39
		Taoism = 1	17	0.14
		Folk beliefs (Mazu, Guan Gong, etc.) = 1	256	2.11
		Islam/Islam = 1	217	1.79
		Catholic = 1	23	0.19
		Christianity = 1	234	1.93
		Other Christia*n =* 1	1	0.01
		Other = None	16	0.13
Time factor	Era	1 in 2018, 0 in 2010	

CGSS2010 and CGSS2018 have the same variable measurement methods in the related questions, the data of CGSS2010 will not be listed in detail.

### 2.4. Model building

#### 2.4.1. Descriptive statistics

The descriptive statistics of the main variables in this paper are shown in Table 5. The dependent variable in the study is sports participation. Regarding the frequency of sports participation, the average value in 2010 was 2.550, which rose to 2.740 in 2018. In the nine-year duration, the enthusiasm of Chinese residents to participate in sports has increased. However, the median of 2010 and 2018 is 2, which means that although the enthusiasm of Chinese residents to participate in sports has increased, it is still not high overall. The standard deviations are 1.721 and 1.747, respectively, which means that there is a big difference in the frequency of residents' sports participation in the two periods.

**Table 5:** Descriptive statistics.

**Variable**	**Mean**		**Median**		**Standard deviation**		**Minimum**		**Maximum value**	
	**2010**	**2018**	**2010**	**2018**	**2010**	**2018**	**2010**	**2018**	**2010**	**2018**
Sports participation	2.550	2.740	2	2	1.721	1.747	1	1	5	5
Household registration	0.603	0.655	1	1	0.490	0.476	0	0	1	1
Gender	0.509	0.548	1	1	0.500	0.498	0	0	1	1
Ethnicity	0.081	0.058	0	0	0.273	0.233	0	0	1	1
Social class	3.037	3.517	3	3	1.838	1.859	1	1	10	10
Family income	10.45	10.93	10.19	11.00	1.703	2.303	7.474	0	16.12	16.12
Age	53.24	59.08	55	62	16.06	15.63	18	18	96	118
Occupation	0.163	0.202	0	0	0.369	0.402	0	0	1	1
Individual income	8.647	8.678	9.393	10.27	3.579	3.958	0	0	16.12	16.12
Education level	8.547	9.145	9	9	3.624	4	0	0	19	19
Marital status	0.829	0.749	1	1	0.376	0.434	0	0	1	1
Religious belief	0.122	0.116	0	0	0.328	0.321	0	0	1	1

The independent variables of this study consist of three dimensions:

(1) Social factors dimension includes household registration, gender, and ethnicity. In 2010, the average household registration was 0.603; that is, 60.3% of the households were registered as urban residents. In 2018, this figure rose to 65.5%, which is generally consistent with China's urbanization process. In 2010, the gender average was 0.509; that is, 50.9% of the respondents were female. In 2018, this figure was 54.8%, and the proportion of female respondents increased slightly. In 2010, the average ethnicity value was 0.081, showing that about 8.1% of the respondents were ethnic minorities, and this figure dropped to 5.8% in 2018.
(2) Family factors dimension includes family income and the social class of the respondents when they were 14 years old. Among them, the average value of social class in 2010 was 3.037; that is, the majority of interviewees believed that their native families belonged to the middle and lower classes. In 2018, this number rose to 3.517, indicating that most interviewees had jumped in class. In 2010, the average value of household income after logarithm removal was 10.45; in 2018, it rose to 10.93, and the standard deviations were relatively large, indicating that the family income of different resident groups varies considerably.
(3) Self-induced factors dimension includes factors related to individuals, such as age, occupation, individual income, education level, marital status, and religious belief. In 2010, the average age was 53.24, and in 2018 it rose to 59.08, reflecting the acceleration of the aging process in China. In terms of occupation, based on social experience and previous research, influenced by the socialist system with Chinese characteristics, people generally believe that occupations that work for the state (or are paid by the state) are more stable. This type of occupation is habitually called *work in the system* in China. In 2010, the average occupation value was 0.163; that is, about 16.3% of the respondents were working in the system. In 2018, this figure rose to 20.2%. For individual income in 2010 and 2018, the mean value after taking the logarithm is 8.647, and the standard deviation is slight different, indicating that the income difference of resident groups has changed little. The average education level in 2010 was 8.547, and it rose to 9.145 in 2018, with a median of 9, which means that the intermediate education level of the interviewed households is about junior high school. The average value of marital status in 2010 was 0.829, indicating that 82.9% of the interviewed households were married; this figure dropped to 74.9% in 2018, which also explained the decline in the marriage registration rate. It should be noted that in statistics, remarriage with spouse is considered as a type of marital change, so this indicator is not included in the statistics of married. Meanwhile, considering that the sample size of remarried with spouse is small (*n* = 200), its impact on the statistical results can be ignored. The average value of religious belief in 2010 was 0.122, indicating that religious believers accounted for 12.2% of the total sample, and this figure dropped to 11.6% in 2018.

#### 2.4.2. Ordered Probit model

The empirical goal of this paper is to explore the influencing factors of Chinese residents' sports participation. Since the dependent variable in this paper is an ordered discrete variable, when the explained variable is a multivariate ordered discrete variable, the ordered Probit model can better meet the needs of empirical regression. Compared with the traditional ordered Logit model, the ordered Probit model relaxes the assumption of the independence of irrelevant alternatives of the sample data and has broader applicability. Therefore, this paper establishes the ordered Probit model. The equations are as follows:


$$\begin{array}{l} Y^{*}=\sum_{i=1}^{5}\alpha_{i}+\beta_{1}urban+\beta_{2}sex+\beta_{3}nation+\beta_{4}rank\\ \;+\beta_{5}fa\_income+\beta_{6}age+\beta_{7}job+\beta_{8}per\_income+\beta_{9}edu\\ \;+\;\beta_{10}marry+\beta_{11}belief+\beta_{12}time+\varepsilon \end{array}$$

Among them, Y^*^ is the frequency of resident sports participation in the dependent variable, which is divided into five grades: “ I do not exercise”, “several times a year or even less”, “several times a month”, “several times a week”, and “exercise every day”. α_i_ is a constant term, there are five categories of dependent variable values, and five constant terms will be generated under the ordered multiple regression. β_i_ is the regression coefficient of the corresponding *i-*th influencing factor, and ε_*i*_is the residual term. Set the threshold λ_1_ < λ_2_ < λ_3_ < < λ_4_ < λ_5_, there is the formula:


$$\begin{array}{l} \text{ 1 }if\;Y^{*}\leq {\text{λ}}_{1}\\ \text{Y}=\text{2 }if\;{\text{λ}}_{1}<Y^{*}\leq {\text{λ}}_{2}\;\\ \text{ 3 }if\;{\text{λ}}_{2}<Y^{\text{*}}\leq {\text{λ}}_{3}\\ \;... \end{array}$$

The probability of *Y* looks like this:


$$\begin{array}{l} Prob(Y=1|X)=Prob(Y^{*}\leq {\text{λ}}_{1}|X)=Prob(X\beta^{T}+\varepsilon_{i})\\ \leq {\text{λ}}_{1}|X=\phi ({\text{λ}}_{1}-X\beta^{T})\;\\ Prob(Y=2|X)=Prob({\text{λ}}_{1}<Y^{*}\leq {\text{λ}}_{2}|X)=\phi ({\text{λ}}_{2}-X\beta^{T})-\phi ({\text{λ}}_{1}-X\beta^{T})\;\\ Prob(Y=3|X)=Prob({\text{λ}}_{2}<Y^{*}\leq {\text{λ}}_{3}|X)=\phi ({\text{λ}}_{3}-X\beta^{T})-\phi ({\text{λ}}_{2}-X\beta^{T})\;\\ Prob(Y=4|X)=Prob({\text{λ}}_{3}<Y^{*}\leq {\text{λ}}_{4}|X)=\phi ({\text{λ}}_{4}-X\beta^{T})-\phi ({\text{λ}}_{3}-X\beta^{T})\\ Prob(Y=5|X)=Prob({\text{λ}}_{4}<Y^{*}\leq {\text{λ}}_{5}|X)=\phi ({\text{λ}}_{5}-X\beta^{T})-\phi ({\text{λ}}_{4}-X\beta^{T}) \end{array}$$

In the above formula, *ϕ* is the standard normal cumulative distribution function.

The study concentrates on the direction and significance of the coefficient *β*_i_ of the independent variable. If it is positive and significant, it means that the factor will promote the increase of the frequency of residents' sports participation; if it is negative and significant, the factor will reduce the frequency of residents' sports participation; if it is insignificant, it means that the factor has no significant relationship with the frequency of residents' sports participation. According to empirical needs, the study will also conduct sub-sample regression on the two-year cross-sectional data in 2010 and 2018 to explore the different changes in the influencing factors in each era.

## 3. Results

### 3.1. Probit regression

According to the sample data, combined with the empirical model established above, an ordered Probit regression is carried out, and robust standard errors are used to overcome the heteroscedasticity problem. The regression results are as follows (see Table 6).

**Table 6:** Benchmark regression.

	**(1)**	**(2)**	**(3)**
	**Full sample**	**Year 2010**	**Year 2018**
Household registration	0.365^***^	0.471^***^	0.332^***^
	(0.044)	(0.100)	(0.049)
Gender	0.057	−0.081	0.088^**^
	(0.035)	(0.079)	(0.039)
Ethnicity	−0.122	−0.256^*^	−0.076
	(0.074)	(0.153)	(0.085)
Social class	0.029^***^	0.002	0.035^***^
	(0.009)	(0.021)	(0.011)
Family income	0.010	0.018	0.007
	(0.008)	(0.021)	(0.009)
Age	0.006^***^	0.012^***^	0.004^***^
	(0.001)	(0.003)	(0.001)
Occupation	0.099^**^	0.129	0.093^*^
	(0.044)	(0.107)	(0.048)
Individual income	0.013^***^	−0.011	0.021^***^
	(0.005)	(0.010)	(0.005)
Education level	0.027^***^	0.046^***^	0.023^***^
	(0.005)	(0.012)	(0.005)
Marriage	0.050	0.082	0.043
	(0.039)	(0.100)	(0.043)
Religious belief	0.002	−0.029	0.011
	(0.053)	(0.129)	(0.059)
Era	0.074^*^		
	(0.042)		
/			
Cut1	1.127^***^	1.404^***^	0.961^***^
	(0.127)	(0.308)	(0.141)
Cut2	1.375^***^	1.650^***^	1.211^***^
	(0.127)	(0.310)	(0.141)
Cut3	1.615^***^	1.805^***^	1.472^***^
	(0.127)	(0.311)	(0.142)
Cut4	1.840^***^	2.177^***^	1.664^***^
	(0.128)	(0.313)	(0.142)
N	0.365^***^	0.471^***^	0.332^***^
R2_p	(0.044)	(0.100)	(0.049)

Standard errors are in brackets,

*** ,

** ,

* represent significant at the 1, 5, and 10% levels, respectively.

As shown in Table 6, in column (1), the study uses a two-year mixed sample of 2010 and 2018 to test the influencing factors of the dependent variable-sports participation. The results show that in terms of social factors, the household registration coefficient is significantly positive at the 1% significance level while gender and ethnicity coefficients are not; that is, urban residents have a higher frequency of sports participation than rural residents, while men and women participate relatively equal in physical exercise. The ethnicity coefficient is insignificant, indicating no noticeable difference in sports preference between Han and ethnic minorities.

Regarding family factors, the coefficient of social class is significantly positive while the coefficient of family income is not, which means that residents of higher social classes are more motivated to participate in sports than residents of lower social classes; and the family income has little effect.

For self-induced factors, age, individual income, education level and the occupation coefficient are all significantly positive, while religious belief and marital status are not; that is, the elderly are more active in physical exercise than the young, which may be because the elderly have sufficient leisure time. It also indicates that residents who work in the system (those who work for the state), residents with high income, and residents with higher education levels are keener to participate in sports.

The era (whether it is 2018) is significantly positive, indicating that the resident' sports participation in 2018 is more active than that in 2010, and it also shows that the residents' sports participation has changed significantly in the 9-year period; ascension is the main feature.

### 3.2. Sub-sample regression

The authors also conduct a sub-sample regression to verify whether the influence of various influencing factors on sports participation has changed from 2010 to 2018, the results can be found in Table 6, columns (2) and (3).

For social factors, the results show that from 2010 to 2018, the coefficient of household registration is still significant, but the size of the coefficient gradually decreases, indicating that despite the current enthusiasm of urban residents to participate in sports is more potent than that of rural areas, but the gap with the latter has been narrowing. Meanwhile, it can be seen that gender was insignificant in 2010 but significantly positive in 2018, indicating that in 2010, the frequency of male and female participation in sports had no significant difference, while after 9 years, female's fitness enthusiasm has improved and been significantly higher than that of male. Also, the ethnicity coefficient changed from significant to negative insignificant; in 2010, compared with the Han group, the overall fitness enthusiasm of ethnic minorities was not strong, and after 9 years, the two were nearly indistinguishable.

For family factors, the coefficient of social class was insignificant in 2010, while it was significantly positive in 2018, which also means that with the evolution of the times, people with higher social status have an increasing awareness of physical fitness, and the frequency of participation has also increased.

For self-introduced factors, the age coefficient was significantly positive in 2010 and 2018, but the coefficient was reduced in 2018, showing that young people's enthusiasm for physical exercise increased with time. The occupation coefficient never increased, which shows that practitioners in the system (those who work for the state) have more enthusiasm for fitness. The individual income coefficient is from insignificant to significantly positive, indicating that groups with high income have a greater increase in fitness enthusiasm. When it turns to education level, its coefficient in 2010 and 2018 were both significantly positive in the middle of the year, but the coefficient shrank significantly in 2018, which means that the frequency of fitness among groups with lower education levels is also increasing.

### 3.3. Robustness check

To verify the validity of the empirical conclusion of the study, that is, whether the empirical conclusion can reveal the correct social phenomenon, or it is only a particular result under the accidental regression, and cannot be generalized as a general conclusion, this paper attempts to conduct a robustness test to check.

Previously, the source of the dependent variable in this paper was the item:

“*B4: How many times a week do you do physical exercise for at least 20 minutes or more? Here refer to those exercises that make sweat or breathe faster.”*

Considering that some sports participants prefer low-intensity exercise without sweating, the author re-selects an item from the questionnaire to replace current dependent variable:

“*A30i. In the past year, did you often engage in the following activities in your spare time-physical exercise?”*

The five grades of “I do not exercise”, “several times a year or even less”, “several times a month “, “several times a week”, and “exercise every day” are, respectively, assigned to 1–5. The intensity is specified, reflecting the degree of participation more comprehensively. Taking this variable as the new dependent variable, the ordered Probit regression is performed again, and the results are shown in Table 7. The sign and direction of the coefficients are still consistent with the previous regression, indicating that the empirical conclusions of this paper are robust.

**Table 7:** Robustness test by replacing the dependent variable.

	**(1)**	**(2)**	**(3)**
	**Full sample**	**Year 2010**	**Year 2018**
Household registration	0.533^***^	0.774^***^	0.479^***^
	(0.042)	(0.102)	(0.047)
Gender	0.087^**^	−0.012	0.108^***^
	(0.034)	(0.080)	(0.038)
Ethnicity	−0.134^*^	−0.241	−0.097
	(0.075)	(0.176)	(0.084)
Social class	0.036^***^	0.060^***^	0.032^***^
	(0.009)	(0.021)	(0.011)
Household income	0.019^**^	0.024	0.018^**^
	(0.008)	(0.020)	(0.008)
Age	0.006^***^	0.014^***^	0.004^***^
	(0.001)	(0.003)	(0.001)
Occupation	0.034	−0.100	0.058
	(0.043)	(0.108)	(0.047)
Individual income	0.009^**^	0.003	0.013^***^
	(0.004)	(0.010)	(0.005)
Education level	0.042^***^	0.048^***^	0.041^***^
	(0.005)	(0.012)	(0.005)
Marriage	0.043	0.047	0.045
	(0.039)	(0.104)	(0.042)
Religious belief	−0.035	−0.111	−0.023
	(0.053)	(0.129)	(0.059)
Era	0.222^***^	
	(0.042)		
/			
Cut1	1.460^***^	2.057^***^	1.104^***^
	(0.122)	(0.311)	(0.135)
Cut2	1.763^***^	2.436^***^	1.391^***^
	(0.122)	(0.312)	(0.135)
Cut3	1.952^***^	2.710^***^	1.564^***^
	(0.123)	(0.315)	(0.135)
Cut4	2.418^***^	3.001^***^	2.068^***^
	(0.124)	(0.318)	(0.136)
N	4870	999	3871
R2_p	0.051	0.088	0.041

Standard errors are in brackets,

*** ,

** ,

* represent significant at the 1, 5, and 10% levels, respectively.

### 3.4. Further analysis

Comparing the two-year data between 2010 and 2018, the influence of most social and self-induced factors shows a certain rigidity. However, the coefficient estimates of the main variables have changed to a certain extent. To test whether the influence of various factors on residents' sports participation has been strengthened or weakened with the evolution of time and social transformation, the authors introduce the multiplication term of each influencing factor and the dummy variable of the era, and conduct an ordered Probit regression analysis again; the results are shown in Table 8.

**Table 8:** Further analysis.

**Social factors**					
**Household registration**	0.548^***^	**Gender**	−0.073	**Ethnicity**	−0.334^**^
	(0.084)		(0.073)		(0.142)
**Era**	0.227^***^	**Era**	−0.010	**Era**	0.055
	(0.075)		(0.060)		(0.043)
Household registration^*^ **era**	−0.232^***^	Gender^*^ **era**	0.162^**^	Ethnicity^*^ **era**	0.280^*^
	(0.089)		(0.081)		(0.164)
**Family Factors**					
**Social class**	0.012		**Family income**	0.139	
	(0.020)			(0.233)	
**Era**	0.006		**Era**	0.015	
	(0.084)			(0.020)	
Social class^*^ **era**	0.022		Family income^*^ **era**	0.139	
	(0.022)			(0.233)	
**Self-induced factors**					
**Age**	0.012^***^	**Occupation**	0.232^**^	**Individual income**	0.000
	(0.002)		(0.099)		(0.010)
**Era**	0.511^***^	**Era**	0.103^**^	**Era**	−0.064
	(0.143)		(0.046)		(0.100)
Age^*^ **era**	−0.008^***^	Occupation^*^ **era**	−0.161	Individual income^*^ **era**	0.016
	(0.003)		(0.108)		(0.011)
**Education level**	0.043^***^	**Marriage**	0.068	**Religious**	−0.086
	(0.010)		(0.096)		(0.120)
**Era**	0.245^**^	**Era**	0.092	**Era**	0.062
	(0.110)		(0.095)		(0.044)
Education level^*^ **era**	−0.019^*^	Marriage^*^ **era**	−0.022	**Religious** ^*^ **era**	0.109
	(0.011)		(0.105)		(0.132)

Standard errors are in brackets,

*** ,

** ,

* represent significant at the 1, 5, and 10% levels, respectively.

For social factors, both household registration and era are significantly positive, but the interaction term between those two is significantly negative, which shows that with the development of the times, the impact of household registration on resident' sports participation is weakening. This result may be related to the reform of the household registration system implemented in China in 2014 and the improvement of rural sports facilities in recent years. According to the *Opinions on Further Promoting the Reform of the Household Registration System* promulgated in 2014, Chinese household registration no longer distinguishes between rural and urban household registration. Compared with rural residents, although urban residents have long-term advantages in sports participation, with the evolution of the times, especially the continuous improvement of rural sports facilities and the awakening of rural fitness awareness, the difference will continue to shrink. On the other hand, the regression results also show that the coefficients of gender and era are not significant, but the interaction term is significantly positive, indicating that although there is no significant difference in the frequency of sports participation between men and women, as time progresses, women's fitness levels are higher than men's. The frequency will be increased to a greater extent. Finally, the regression results show that the ethnicity coefficient is significantly negative, while its interaction term with the era is significantly positive, indicating that compared with the Han, ethnic minorities have a lower frequency of sports participation, but with the evolution of the times, ethnic minorities catch up effect is more pronounced.

For family factors, the regression results show that the coefficient of the interaction term between social class and era is significantly positive, which means that under the blessing of time, groups with a higher social class will be more active in sports. On the other hand, family income, era and their interaction items are insignificant, indicating that family income is not a critical indicator affecting sports participation, and the impact of family income on sports participation will not change with the evolution of the times.

As for self-induced factors, although times are changing, these are still the most crucial aspect affecting residents' sports participation. According to the regression results, the coefficients of age and era are both significantly positive, while the interaction term is significantly negative, indicating that the elderly have a higher frequency of sports participation than the young. Still, as the times evolve, young people will also invest more time in physical exercise, and the inter-generational age gap in sports participation will continue to narrow. At the same time, the occupation and era coefficients are both significantly positive, but the interaction term between the two is insignificant, indicating that the group *working in the system* is more active in participating in physical exercise than the group *working outside the system*. The difference tends to shrink, but the trend is not apparent, indicating that the differences in sport participation in and outside the system will persist for a considerable time. Moreover, the education level and era coefficients are both significantly positive, but the interaction term is significantly negative, indicating that the group with a higher education level has a higher frequency of sports participation than the group with a lower education level. However, with the evolution of the times, the latter will pay more and more attention to their health and spend more time participating in physical exercise, and the inter-generational gap in the education level of sports participation will continue to narrow. On the other hand, individual income, religious belief, and marital status do not affect the frequency of sports participation. Its interaction items are also insignificant, indicating that these characteristics are not the decisive factors affecting sports participation, and the evolution of the times will not significantly impact the change's effectiveness.

## 4. Discussion

### 4.1. Findings

The specific issue under investigation here is whether mass sports participation behavior appears to be characterized by inequities in terms of social, family, and self-induced factors. Using the two-year sample data of CGSS 2010 and 2018, the authors screened 4940 valid observation samples and conducted an ordered Probit regression in Stata 17 software. The empirical results are studied and the following five conclusions are found.

First, in terms of social factors, urban residents have a higher frequency of sports participation than rural residents.

Second, in terms of family factors, residents with higher social classes are more likely to participate in sports than those with lower social classes.

Third, in terms of self-induced factors, the elderly are more motivated to exercise than the young; residents who work in the system (those who work for the state), residents with high incomes, and residents with higher education levels are keener to participate in sports.

Fourth, Chinese residents' mass sports participation rate has generally shown an upward trend over time.

Last but not least, after the robustness test of the results to verify its validity, the study finds that with time changes, the sports participation rate varies between urban and rural areas, between ethnic minorities and Han ethnic, between old and young age groups, between higher and lower education levels will continue to shrink, but differences between social classes will further increase over time.

### 4.2. Limitations

Since the study used ordered Probit regression to discuss the influencing factors and changing trends of Chinese residents' sports participation, the author's interpretation of the economic significance of the variable coefficient is mainly based on the current situation and previous research results. It cannot conduct a further in-depth discussion on the impact mechanism.

As shown above, the study also used sub-sample regression to illustrate the changing trends and effects of time. Sub-sample test is a classic method in social science and has been used for a long time, which may not be fashionable enough. Since we have already added interaction terms to our variables in Probit analysis, it is meaningless to multiply variables by interaction terms again, so we still chose sub-sample regression. Nevertheless, to ensure the results' robustness, we also replaced the dependent variable as a supplement in the robustness check section; the results are meaningful and robust. Therefore, we hope future researchers could dig out better and more fashionable methods to replace the sub-sample regression.

Moreover, considering that the study focused on the influencing factors of sports participation rather than discussing a specific independent variable's influence on the dependent variable, the authors carefully selected the sample and adopted a method of replacing the dependent variables to reduce the effect of endogeneity. However, the study cannot deny that there exist more influencing factors in real society other than the variables covered by design, and the endogeneity could not essentially avoid. We can only guarantee the mentioned factors have been thoroughly explained and proven to have a non-negligible impact.

Hence, based on the limitations, the authors hope that future research could design models that include a more comprehensive range of indicators so that discussions on mass sport participation can get further deepened.

## 5. Conclusion

This paper explores the influencing factors of residents' sports participation, analyzes the role of social, family, and self-induced factors in residents' mass sports participation, and studies the temporal trend of the above factors.

It can be concluded that inequality existed in the access to mass sports on social, family, and self-induced factors. Suppose China and other developing countries would like to reduce the inequality of sports participation caused by social and family factors such as the urban-rural gap and social class disparity, they need to increase investment in rural public sports facilities and fundamentally change residents' awareness of healthy lifestyles.

Meanwhile, considering that self-induced factors are the most critical factors affecting the inequality of mass sports participation, improving the educational level and individual income can also reduce the gap. On the educational aspect, China has implemented a 9-year compulsory education policy since 1986; as of 2018, the average length of educated duration in China is maintained at about nine years, which is very low. Over the past 30 years, given that residents' income levels and educational concepts have changed tremendously, policymakers may consider extending compulsory education to 12 years as most developed countries do. On the individual income aspect, it should distinguish individual income and family income first. Individual income measures the individual's economic level, while family income reflects the family's survival. Although China's economy has developed rapidly in recent years, the fact that the agricultural population accounts for the majority has not changed fundamentally. Measurements based on family statistics need to consider the number of hidden unemployed or non-labor force households in Chinese society. Based on this situation, the relative advantage of the individual income will disappear, because the individual has the obligation and responsibility to help other family members financially. That is to say, the higher overall income of individuals does not mean that the family's total income is also higher. This situation should be more common in developing countries. Therefore, while paying attention to improving personal income, policymakers need to consider the ultimate impact of policies on personal benefits separately.

Inequality of social class will bring socio-political and economic instability. Although mass sports participation is only one indicator of a healthy lifestyle, if the country continues to ignore equal access to mass sports participation, it will eventually affect the stability of the social structure.

As a developing country, China has already reached the upper middle-income status by World Bank definitions. Most developing countries are growing much slower than China and remain relegated to low-income or lower-middle-income status. Based on this consideration, inequality in mass sports participation in China can be seen as an inevitable process that other developing countries will experience in the future, and the discussion of the study using China as a case can provide prior experience to a certain extent. However, China's political system and economic development process differ greatly from most developing countries. Developing countries should fully understand and analyze their national conditions when learning from China's experience.

## Data availability statement

Publicly available datasets were analyzed in this study. This data can be found here: http://www.cnsda.org/index.php?r=projects/index.

## Ethics statement

Ethical review and approval was not required for the study on human participants in accordance with the local legislation and institutional requirements. The patients/participants provided their written informed consent to participate in this study.

## Author contributions

YW conceived the study, collected and analyzed the data, and prepared the manuscript draft. HD provided comprehensive editing, interpretation of the data, and manuscript refinement. WL sorted out the literature. JD put forward suggestions for revision. All authors read and approved the final manuscript.
